# isomiRTar: a comprehensive portal of pan-cancer 5′-isomiR targeting

**DOI:** 10.7717/peerj.14205

**Published:** 2022-10-17

**Authors:** Stepan Nersisyan, Aleksandra Gorbonos, Alexey Makhonin, Anton Zhiyanov, Maxim Shkurnikov, Alexander Tonevitsky

**Affiliations:** 1 Shemyakin-Ovchinnikov Institute of Bioorganic Chemistry, Russian Academy of Sciences, Moscow, Russia; 2 Institute of Molecular Biology, The National Academy of Sciences of the Republic of Armenia, Yerevan, Armenia; 3 Armenian Bioinformatics Institute (ABI), Yerevan, Armenia; 4 Faculty of Biology and Biotechnology, HSE University, Moscow, Russia; 5 Art Photonics GmbH, Berlin, Germany

**Keywords:** IsomiR, MiRNA, TCGA, Web portal

## Abstract

Inaccurate cleavage of pri- and pre-miRNA hairpins by Drosha and Dicer results in the generation of miRNA isoforms known as isomiRs. isomiRs with 5′-end variations (5′-isomiRs) create a new dimension in miRNA research since they have different seed regions and distinct targetomes. We developed isomiRTar (https://isomirtar.hse.ru)—a comprehensive portal that allows one to analyze expression profiles and targeting activity of 5′-isomiRs in cancer. Using the Cancer Genome Atlas sequencing data, we compiled the list of 1022 5′-isomiRs expressed in 9282 tumor samples across 31 cancer types. Sequences of these isomiRs were used to predict target genes with miRDB and TargetScan. The putative interactions were then subjected to the co-expression analysis in each cancer type to identify isomiR-target pairs supported by significant negative correlations. Downstream analysis of the data deposited in isomiRTar revealed both cancer-specific and cancer-conserved 5′-isomiR expression landscapes. Pairs of isomiRs differing in one nucleotide shift from 5′-end had poorly overlapping targetomes with the median Jaccard index of 0.06. The analysis of colorectal cancer 5′-isomiR-mediated regulatory networks revealed promising candidate tumor suppressor isomiRs: hsa-miR-203a-3p—+1, hsa-miR-192-5p—+1 and hsa-miR-148a-3p—0. In summary, we believe that isomiRTar will help researchers find novel mechanisms of isomiR-mediated gene silencing in different types of cancer.

## Introduction

MiRNAs are short non-coding molecules that post-transcriptionally regulate gene expression (Hobert, 2008). A mature miRNA associated with a member of the Argonaute proteins family directly binds to a target mRNA, which results in degradation of the mRNA or translation repression (Nilsen, 2007). It is known that most of such interactions require base pairing of a miRNA seed region (six nucleotides located at positions 2-7 from the miRNA 5′-end) with a target mRNA (Bartel, 2009; Agarwal et al., 2015). Aberrant miRNA expression and targeting were shown to play an essential role in various pathologies, including different types of cancer (Garzon, Calin & Croce, 2009; Visone & Croce, 2009).

Previously analyzed miRNA-seq reads revealed the existence of nucleotide sequence variability at 5′- and 3′-ends of mature miRNAs; these variants of miRNAs were termed isomiRs (Morin et al., 2008). The main pathway of isomiR biogenesis is related to the miRNA hairpin cleavage by Drosha and Dicer enzymes: because of imprecise cleavage, mature miRNAs could differ from each other by the presence or absence of several nucleotides at their 5′- or 3′-termini (Neilsen, Goodall & Bracken, 2012). We recently showed that almost all miRNAs are heterogeneous at their 3′-ends, while inaccurate Dicer cleavage was observed at 42% of miRNA 5′-ends, and inaccurate Drosha 5′-end cleavage was the rarest case (14% of miRNAs) (Zhiyanov, Nersisyan & Tonevitsky, 2021).

Since a seed region of a miRNA is located at positions 2-7, even a single nucleotide length variation at the 5′-end could alter the seed sequence, hence altering the targetome of the miRNA. While this is easy to confirm with bioinformatics sequence-based target prediction tools, only a few studies supported this phenomenon by an experiment. Namely, 5′-isomiR overexpression followed by the luciferase reporter assay analysis validated differential targeting for different 5′-isomiRs of miR-101 (Llorens et al., 2013), miR-9 (Tan et al., 2014), miR-183 (Telonis et al., 2015), miR-34/449 (Mercey et al., 2017) and miR-411 (van der Kwast et al., 2020). However, the role of isomiRs in cancer remains to be uncovered.

To the best of our knowledge, no publicly available databases summarize the results of computational isomiR target prediction. The situation is additionally complicated because most of the existing bioinformatics tools are not suited for large-scale custom target prediction required for non-canonical isomiRs. Given that, we developed isomiRTar (https://isomirtar.hse.ru)–the web portal which allows one to explore 5′-isomiR targeting in 31 cancer types from the Cancer Genome Atlas (TCGA). First, we predicted targets of all 5′-isomiRs by two widely used bioinformatics tools: miRDB (Chen & Wang, 2020) and TargetScan (Agarwal et al., 2015). Then, the interactions between 5′-isomiRs and putative targets were subjected to co-expression analysis in each cancer type. This resulted in the interactions supported by a significant negative correlation in cancer samples. Aside from the web portal implementation, we also analyzed general properties of 5′-isomiR targeting (such as the impact of one nucleotide variation on a targetome) and explored the colorectal cancer 5′-isomiR-gene regulatory network in depth.

## Materials & Methods

### TCGA data collection and processing

RNA-seq and miRNA-seq read count tables for primary solid tumor samples were downloaded from the GDC portal (https://portal.gdc.cancer.gov/). Thirty-one TCGA projects were selected for the downstream analysis (Table S1). First, library size normalization of RNA-seq data was independently conducted for each cancer type using edgeR TMM algorithm implementation (Robinson, McCarthy & Smyth, 2010), and the default low-expressed gene removal procedure was applied. After normalizing for transcript length, TMM-normalized fragments per kilobase of transcript per million mapped reads (TMM-FPKM) tables were generated. Finally, TMM-FPKM tables were ommllog2-transformed.

Processing of miRNA-seq data was started from the 5′-isomiR annotation from *.mirbase21.isoforms.quantification.txt files. For that, pri-miRNA genomic coordinates and local coordinates of canonical mature miRNA sequences were extracted from miRBase v21 (Kozomara, Birgaoanu & Griffiths-Jones, 2019). The standard 5′-isomiR nomenclature was utilized: a number after the “—” character denotes a shift from the canonical 5′-end in the 5′-3′ direction. For example, hsa-miR-192-5p—+1 differs from the canonical hsa-miR-192-5p miRNA by the absence of the first nucleotide on its 5′-end. As in the case of mRNA expression data, the edgeR package was used to filter out low expressed isomiRs and generate ommllog2-transformed TMM-normalized reads per million mapped reads (TMM-RPM) tables. It is well known that the overwhelming majority of miRNA sequencing reads correspond to the dozens of the most highly expressed isomiRs (Shao et al., 2010; Nersisyan et al., 2020a). Given that we marked a minimal set of 5′-isomiRs accounting for 95% of sequencing reads in a TCGA project as *highly expressed* (Table S1). A typical read count distribution is presented in Fig. S1 for the TCGA-COAD project.

### Sequence- and correlation-based analysis of 5′-isomiR targeting

IsomiR target prediction was conducted with two commonly used tools: miRDB v6.0 (Chen & Wang, 2020) and TargetScan v7.2 (Agarwal et al., 2015).

The custom prediction mode of the miRDB web portal (http://mirdb.org) was programmatically called for each isomiR sequence using the Requests module of Python. The default 80 threshold value was set for miRDB target scores to select reliable interactions. Due to the technical limitations in miRDB, target prediction was performed only for isomiRs with a sequence length between 17 and 29 nucleotides.

Publicly available TargetScan Perl scripts and UTR data were obtained from the official web portal (https://www.targetscan.org/vert_72/). Dr. George Bell kindly provided Perl scripts for calculating cumulative weighted context++ scores (CWCS). TargetScan predictions almost always covered miRDB predictions (see Results). Because of that, and since there is no conventional CWCS cut-off value, we equalized the numbers of miRDB/TargetScan predicted targets for each 5′-isomiR. Specifically, for each 5′-isomiR we sorted the predictions according to the CWCS (lowest to highest) and then selected top *n* entries of the list, where *n* is the number of targets of this isomiR predicted by miRDB. Finally, filtered miRDB and TargetScan predictions were merged.

For a pair of 5′-isomiR and predicted target, we calculated Spearman’s correlation coefficient *r* between corresponding expression levels in each cancer type. The following thresholds were used to define the significance of interaction: *r* <  − 0.3, adjusted *p*-value <0.05. The same standard criteria for considering miRNA-target correlation significant was previously used in many works (Hu et al., 2016; Paquette et al., 2019; Nersisyan et al., 2020b). Multiple testing correction was performed differently depending on the analysis purpose:

1. Analysis of a cancer-specific isomiR-target regulatory network. In this case, the Benjamini–Hochberg procedure was applied to the list of *p*-values corresponding to all putative isomiR-target interactions (referred to as FDR_global_ in the isomiRTar interface).
2. Analysis of specific 5′-isomiR targets or specific target gene regulators in a fixed cancer type (out of other interactions context). In this case, the Benjamini–Hochberg procedure was applied only to the narrow list of corresponding *p*-values (referred to as FDR_local_ in the isomiRTar interface).

### Functional annotation analysis

Functional annotation analysis of gene sets was performed with DAVID 2021 (Sherman et al., 2022). The default 0.05 cut-off value for FDR was used to select significantly enriched KEGG pathways.

### Network and statistical analyses, web portal implementation

Networks were constructed and analyzed using the NetworkX v2.8 Python module (Hagberg, Schult & Swart, 2008). Maximal cliques of isomiR-isomiR co-expression networks were found with the find_cliques function.

SciPy v1.5 Python module (Virtanen et al., 2020) was used for the statistical analysis. Pandas v1.3 (McKinney, 2010), NumPy v1.19 (Harris et al., 2020), Seaborn v0.11 (Waskom, 2021), and Matplotlib v3.5 (Hunter, 2007) Python modules were extensively used for the miscellaneous computations and visualization.

Implementation of the isomiRTar website was based on Flask v2.0 Python framework and PostgreSQL database. All isomiRTar source codes have been made available on GitHub (https://github.com/s-a-nersisyan/isomiRTar).

## Results

### Pan-cancer expression landscape of 5′-isomiRs

**Figure 1:**
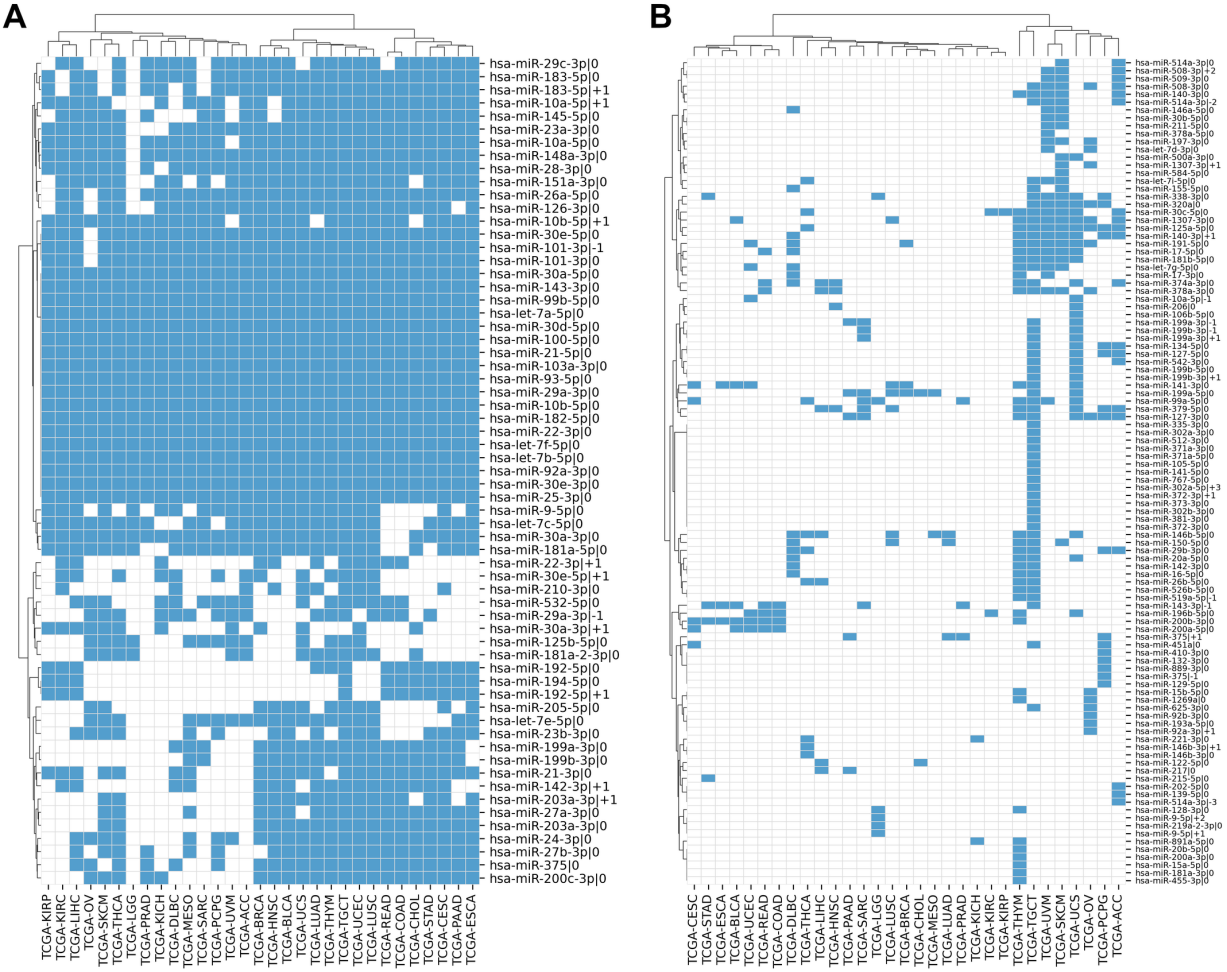
Pan-cancer expression distribution of 5′-isomiRs. (A) Binarized expression profiles of 63 abundantly expressed 5′-isomiRs (highly expressed in ten or more cancer types). (B) Binarized expression profiles of 105 cancer-specific 5′-isomiRs (highly expressed in less than 10 cancer types). Blue cells stand for highly expressed 5′-isomiRs.

We compiled the comprehensive list of 1022 5′-isomiRs expressed in 31 cancer types using TCGA miRNA sequencing data. Out of them, 168 5′-isomiRs were marked as highly expressed in at least one cancer type, which included 31 non-canonical isomiRs. Approximately 38% of highly expressed isomiRs (63 entries) were expressed in 10 or more cancer types, including 11 non-canonical 5′-isomiRs: hsa-miR-10a-5p—+1, hsa-miR-10b-5p—+1, hsa-miR-22-3p—+1, hsa-miR-29a-3p—-1, hsa-miR-30a-3p—+1, hsa-miR-30e-5p—+1, hsa-miR-101-3p—-1, hsa-miR-142-3p—+1, hsa-miR-183-5p—+1, hsa-miR-192-5p—+1, hsa-miR-203a-3p—+1. Finally, 18 miRNAs (including almost all representatives of let-7 and miR-30 families) were expressed at high levels in all 31 TCGA projects (Fig. 1A). The rest of 5′-isomiRs (105 entries, 62%) were expressed in a cancer-specific manner (less than 10 cancers, Fig. 1B).

In most cases, the high expression of a non-canonical 5′-isomiR was tied with the high expression of its corresponding canonical form. There were only two exceptions: the canonical forms of hsa-miR-302a-5p—+3 and hsa-miR-519a-5p—-1 (both were highly expressed in TCGA-TGCT) were not detectable in the analyzed data, suggesting “incorrect” miRBase annotation or systematical sequencing biases. Interestingly, several miRNAs had three highly expressed 5′-isomiRs: hsa-miR-10a-5p (—-1, —0, —+1), hsa-miR-199a-3p (—-1, —0, —+1), hsa-miR-199b-3p (—-1, —0, —+1), hsa-miR-375 (—-1, —0, —+1), hsa-miR-514a-3p (—-3, —-2, —0) and hsa-miR-9-5p (—0, —+1, —+2).

### Large-scale sequence-based prediction of 5′-isomiR target genes

**Figure 2:**
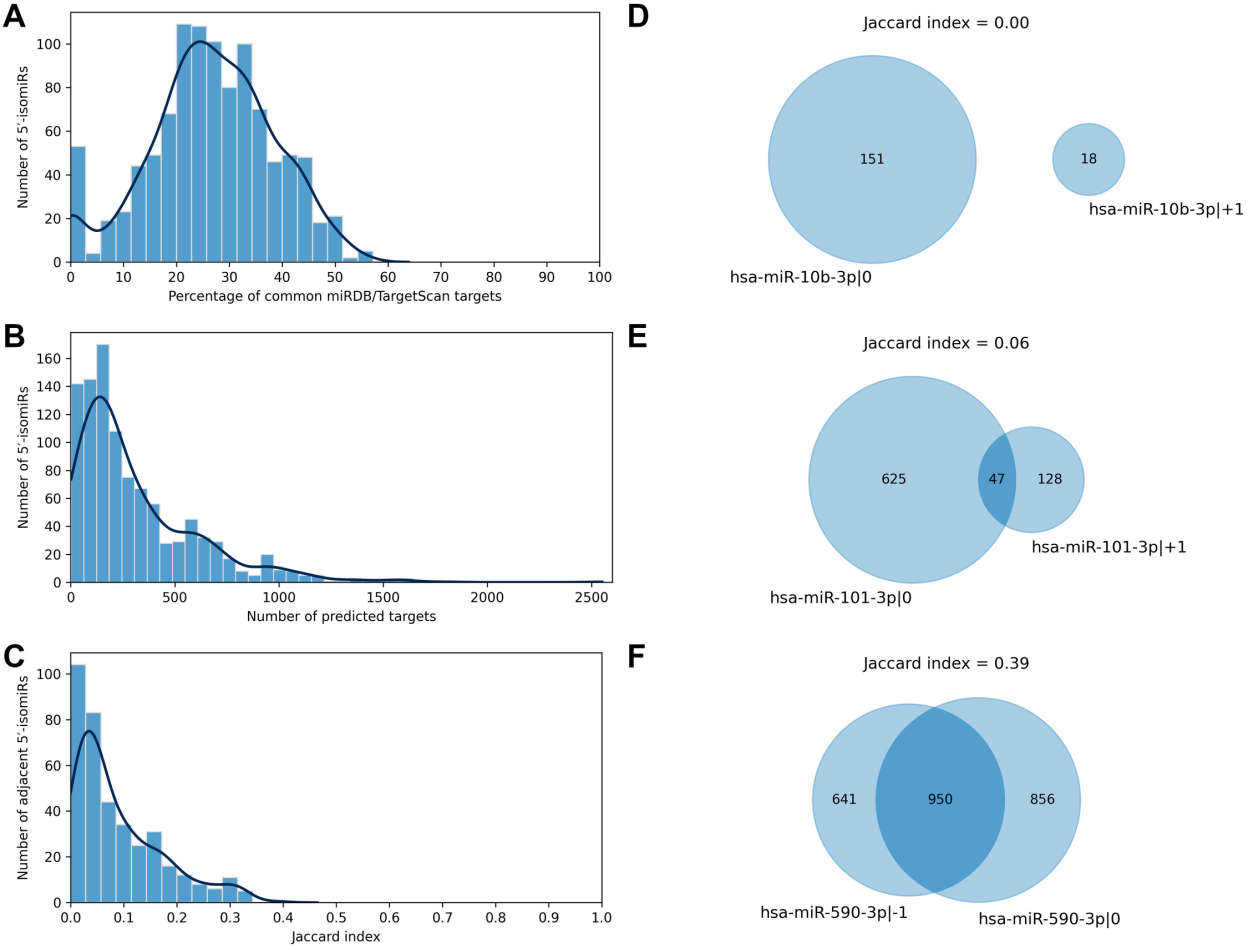
Predicted targets of 5′-isomiRs. (A) Distribution of the percentage of common miRDB/TargetScan targets. (B) Distribution of the number of predicted 5′-isomiR targets. (C) Distribution of the Jaccard indices calculated for the sets of predicted targets for the pairs of adjacent 5′-isomiRs. (D–F) Targetomes of the representative adjacent 5′-isomiRs.

We applied two state-of-the-art tools (miRDB and TargetScan) to predict target genes for all considered 5′-isomiRs. The tools are based on different ideas: the miRDB machine learning algorithm was trained on RNA-seq data of HeLa cells transfected with 25 miRNA mimics to predict miRNA targets based on binding site sequence and structure (Liu & Wang, 2019). TargetScan algorithm also scores miRNA bindings sites according to their cross-species conservation (Agarwal et al., 2015). TargetScan systematically yielded a significantly higher number of isomiR targets than miRDB, and the majority of miRDB targets were included in TargetScan predictions: median 95.5% with 95% confidence interval (CI) 85.7–100.0%. To harmonize these data, we equalized the numbers of predicted targets for each 5′-isomiR by selecting the respective number of the best-ranked targets according to TargetScan (see Materials and Methods for details). After such a harmonization, the predictions of miRDB and TargetScan had a median of 26.5% common targets, 95% CI [0.0–48.9]% (Fig. 2A). From here onwards, we consider the union of predictions by miRDB and TargetScan for each 5′-isomiR, unless indicated otherwise.

The number of sequence-based predicted targets significantly varied between 5′-isomiRs. The median number of targets was 209, and 95%-CI was wide: 6-1082 (Fig. 2B). The canonical form of hsa-miR-3613-3p had the highest number of putative target genes (2551), while five isomiRs had no targets which passed the defined predictions significance thresholds: hsa-miR-1307-5p—0, hsa-miR-1307-3p—+2, hsa-miR-1307-3p—0, hsa-miR-1307-3p—+1 and hsa-miR-371a-3p—+2.

We used the sequence-based predictions to assess the impact of one nucleotide difference in a miRNA 5′-end on its targetome. For each pair of 5′-isomiRs that differ in one nucleotide shift from the 5′-end (*adjacent 5*′*-isomiRs*), we calculated the Jaccard index (JI) for the sets of putative targets—the size of the intersection divided by the size of the union of the predicted target sets (Gösgens et al., 2021). JI = 1 indicates the complete equality of the target sets, JI = 0 indicates that the target sets are disjoint and intermediate values between 0 and 1 indicate the degree of the overlap. For example, the Jaccard index for two 5′-isomiRs with 20 common and 60 targets each equals 20/(60 + 60 − 20) = 0.2. Surprisingly, the median Jaccard index was equal to 0.06, 95% CI [0.00–0.31], indicating the average poor intersection between targetomes of adjacent 5′-isomiRs (Fig. 2C). Thus, different 5′-isomiRs originating from the same hairpin can generally be considered as independent functional units. Representative pairs of adjacent 5′-isomiRs are shown in Fig. 2: hsa-miR-10b-3p—0, hsa-miR-10b-3p—+1 (no common targets, Fig. 2D), hsa-miR-101-3p—0, hsa-miR-101-3p—+1 (median overlap with JI = 0.06, Fig. 2E) and hsa-miR-590-3p—-1, hsa-miR-590-3p—0 (maximum overlap with JI = 0.39, Fig. 2F). Next, we searched for nucleotide motifs associated with high values of JI. For that, 6-mer seed regions of the adjacent 5′-isomiRs were merged into 7-mer sequences (*merged seed regions*). Then, duplicate merged seed regions originating from closely related mature miRNAs (*e.g.*, hsa-miR-19a-3p and hsa-miR-19b-3p) were removed. It turned out that 49 out of 50 merged seed regions corresponding to 50 adjacent 5′-isomiRs with the highest JI values started with uracil (*χ*^2^ test *p* = 1.29 × 10^−30^). Interestingly, we did not find any motifs for the seed sequences of adjacent 5′-isomiRs with non-overlapping targetomes.

### Pan-cancer correlation analysis of 5′-isomiRs and their target genes

The conducted sequence-based target prediction allowed us to compare the putative non-cancer-specific targetomes of 5′-isomiRs. To determine which predicted targets could be downregulated by the corresponding 5′-isomiRs on transcriptome level, we selected isomiR-gene interactions with a significant negative correlation of corresponding expression levels in a set of TCGA samples (the analysis was separately performed for each cancer type). As a result, we could construct the 5′-isomiR-gene interaction networks for each cancer from TCGA. For a given 5′-isomiR and cancer type, we denote the number of significantly anti-correlated 5′-isomiR targets (*i.e.,* the out-degree) as *5*′*-isomiR targeting activity (ITA)*.

**Figure 3:**
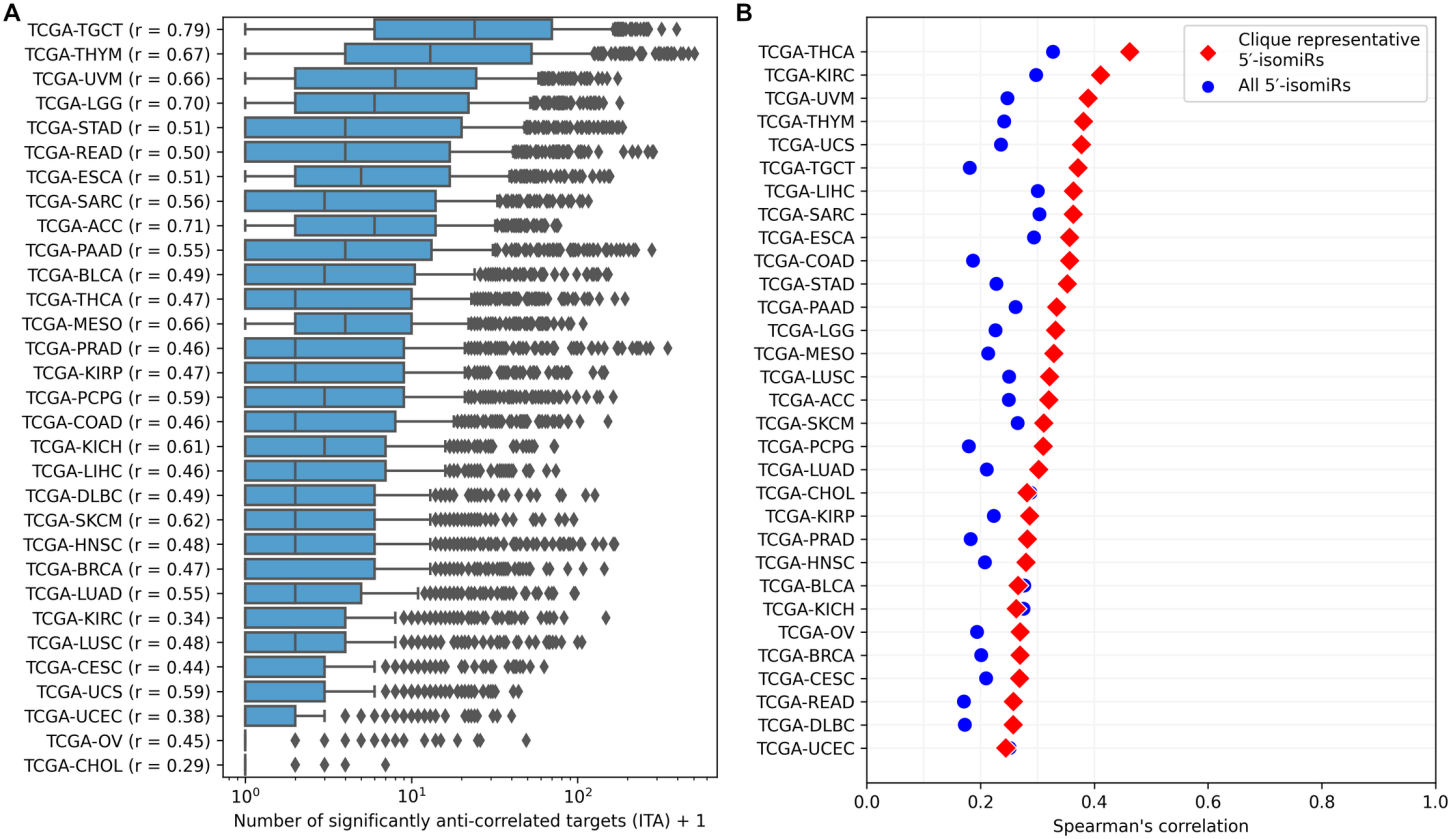
Pan-cancer 5′-isomiR targeting activity (ITA) analysis. (A) Distribution of ITAs across different cancer types. The vertical axis labels contain Spearman’s correlation coefficients between ITAs and numbers of sequence-based predicted targets. (B) Distribution of Spearman’s correlation coefficients between ITAs and median 5′-isomiR expression levels.

ITA values substantially varied between cancer types, but generally, the median ITA did not exceed 10 (Fig. 3A), which was an order of magnitude lower than the median number of sequence-based predicted targets. Nevertheless, there were several isomiRs with broad functional activity (ITA >100) in almost all cancers. The number of sequence-based predicted targets was the primary determinant of ITA: the corresponding Spearman’s correlation coefficients were all positive and significantly different from zero for all cancers (see the vertical axis labels in Fig. 3A). Interestingly, these coefficients strongly varied between cancer types from 0.29 (TCGA-CHOL) to 0.79 (TCGA-TGCT) and were significantly correlated with the median ITA values (Spearman’s correlation = 0.8, *p* = 4.71 × 10^−8^).

Expression levels of 5′-isomiRs also positively and statistically significantly correlated with ITA for all cancer types (*p* < 10^−3^), though correlation coefficients were not high (between 0.2 and 0.3, Fig. 3B). We hypothesized that some low-expressed 5′-isomiRs had high ITA because of a co-expression with other active molecules. Given this, we first identified 5′-isomiR co-expression modules in each cancer type. Modules were defined as maximal cliques in corresponding co-expression graphs, where two isomiRs were connected with an undirected edge if absolute Spearman’s correlation between their expression levels exceeded 0.5. Then, a representative 5′-isomiR with the highest median expression level was selected from each module. Limiting the analysis to these representative isomiRs systematically increased the correlation values between ITA and average expression in all except two cancer types (Fig. 3B). Notably, there was a complete absence of correlation between ITA and expression in the set of highly expressed 5′-isomiRs in each cancer, indicating that high expression is a necessary but insufficient condition for the downregulation of multiple target genes. The same holds for 18 miRNAs with high expression in all 31 cancer types: ITA values varied from less than ten anti-correlated targets (hsa-miR-100-5p, hsa-miR-99b-5p, hsa-miR-10b-5p) to dozens and hundreds (Fig. S2). Indeed, low values of ITA do not imply the absence of isomiR activity, *e.g.*, in the case of very target-specific regulation.

### Overview of isomiRTar portal

To make the 5′-isomiR pan-cancer expression profiles and the results of targeting analysis easily accessible, we developed the isomiRTar web portal (https://isomirtar.hse.ru). The portal’s main page contains the forms for the five query modes: by cancer type, by 5′-isomiR, by gene, by 5′-isomiR, gene, and cancer triple.

**Figure 4:**
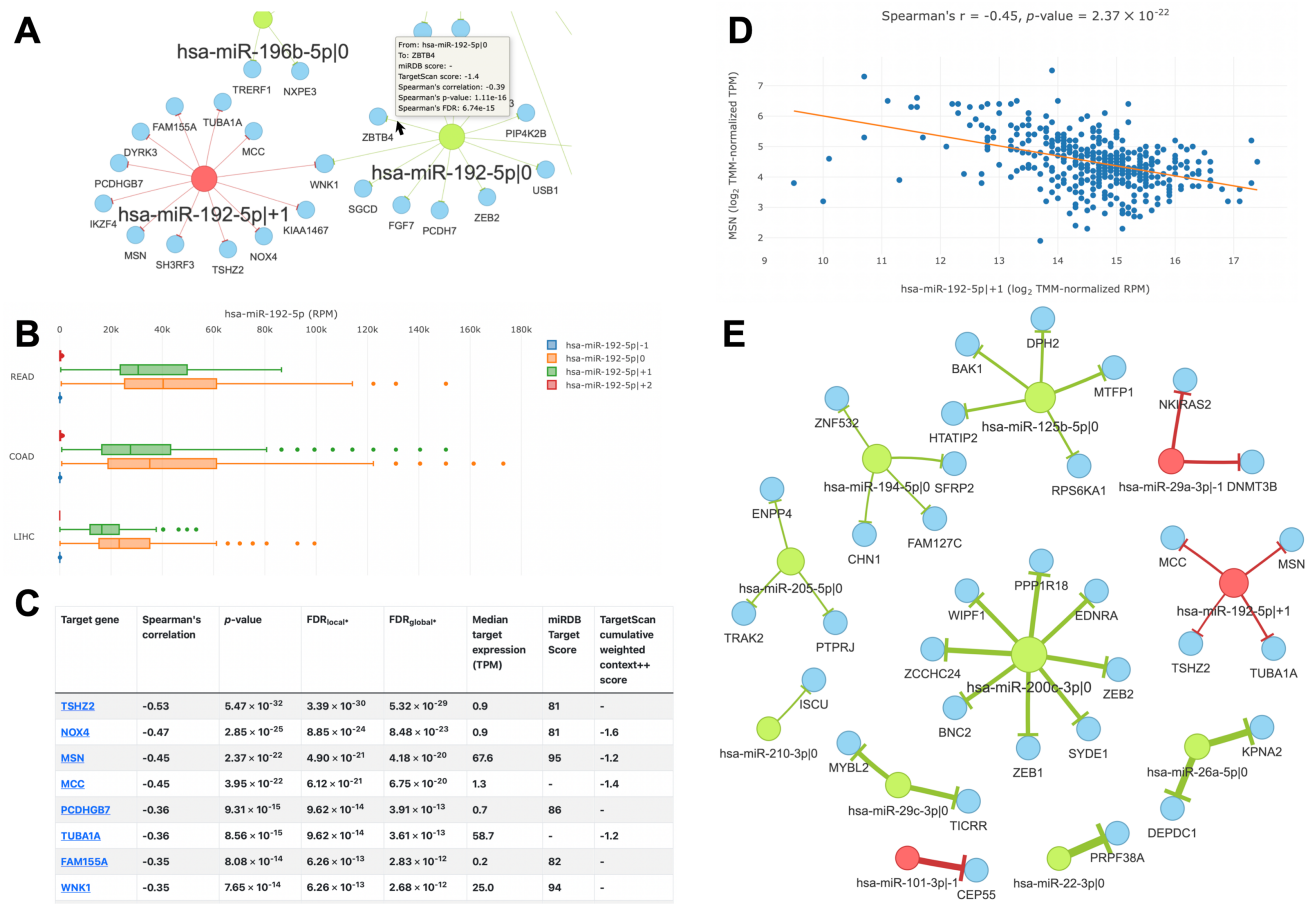
Overview of the isomiRTar portal. (A) The fragment of the colorectal cancer interaction network. (B) The pan-cancer expression distribution of hsa-miR-192-5p 5′-isomiRs. (C) Target prediction and correlation analysis results for hsa-miR-192-5p—+1 in colorectal cancer. (D) The joint expression distribution plot for hsa-miR-192-5p and its target gene MSN in colorectal cancer. (E) The network of “universal” (not cancer-specific) isomiR-gene interactions. Green nodes represent canonical 5′-isomiRs, red nodes represent non-canonical 5′-isomiRs, and blue nodes represent target genes. Edge colors are inherited from adjacent 5′-isomiRs. The size of a node linearly depends on its out-degree. The width of an edge linearly depends on number of cancers with significant negative correlation.

The main feature of the “query by cancer” page is an interaction network between 5′-isomiRs and their target genes in a specified cancer type. As the network is highly interactive, a user can retrieve information about a 5′-isomiR, target gene, or their interaction by simply hovering over corresponding nodes or edges (Fig. 4A). Aside from the graphical representation, the page contains summary statistics for 5′-isomiRs in a selected cancer: median expression, number of predicted targets, number of predicted targets supported by the significant negative correlation. The second query type allows one to explore a pan-cancer expression profile for all 5′-isomiRs of a specified miRNA (Fig. 4B).

The analysis of targets for a given 5′-isomiR or 5′-isomiR regulators of a given mRNA can be performed in two modes: cancer-specific or pan-cancer. In the first case, the portal page contains a table with the results of correlation analysis between a selected 5′-isomiR and its predicted targets or predicted 5′-isomiR regulators and a selected target gene (Fig. 4C). By clicking to interaction, a user can see the scatterplot of corresponding expression values (Fig. 4D).

In the second case (pan-cancer mode), isomiR-target interactions are sorted according to the number of cancers, where we observed the significant anti-correlation between corresponding expression levels. Thus, “universal” (not cancer-specific) isomiR-gene interactions can be inferred from isomiRTar. To analyze the landscape of such “universal” interactions, we selected abundantly expressed 5′-isomiRs (highly expressed in 10 or more cancer types) and incident interactions, which were supported by significant negative correlations in at least half of cancers where the corresponding 5′-isomiR was highly expressed. The resulting network is illustrated in Fig. 4E. Aside from crucial and extensively studied interactions such as the downregulation of ZEB1 and ZEB2 by miR-200 (Hill, Browne & Tulchinsky, 2013; Nersisyan et al., 2021b), the network had several regulatory interactions mediated by non-canonical 5′-isomiRs: hsa-miR-29a-3p—-1, hsa-miR-101-3p—-1 and hsa-miR-192-5p—+1. The regulation of centrosomal protein 55 (CEP55) by hsa-miR-101-3p—-1 was the most pan-cancer conserved: a significant negative correlation was observed in 15 cancer types. Since CEP55 is a well-studied oncogene (Jeffery et al., 2016), this 5′-isomiR of hsa-miR-101-3p acted as a potential tumor suppressor, which agrees with the tumor suppressor role of the canonical hsa-miR-101-3p form (Wang et al., 2018).

### The interaction network of 5′-isomiRs and their targets in colorectal cancer

**Figure 5:**
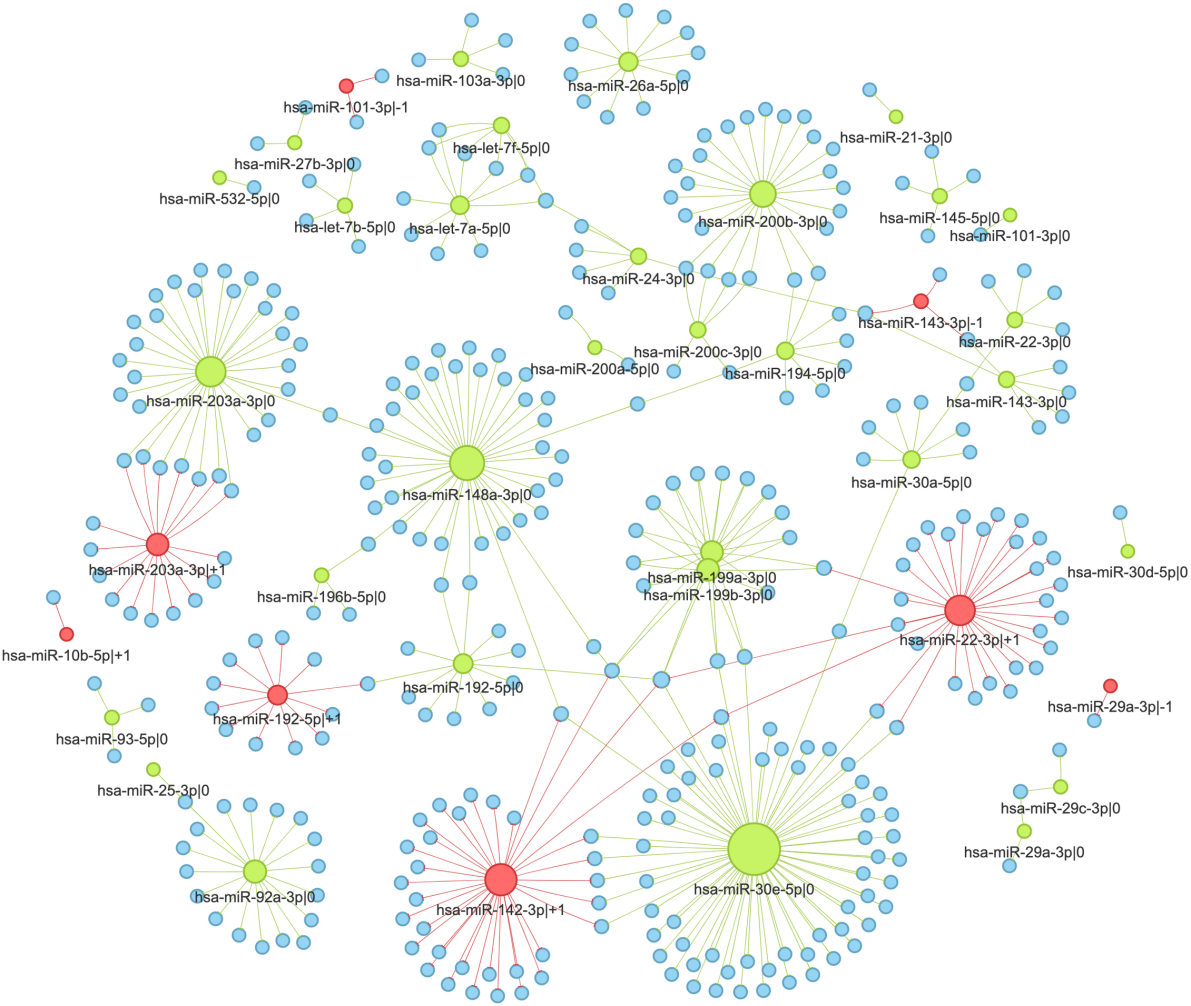
Interaction network of highly expressed 5′-isomiRs and their target genes in colorectal cancer. Green nodes represent canonical 5′-isomiRs, red nodes represent non-canonical 5′-isomiRs, and blue nodes represent target genes. Edge colors are inherited from adjacent 5′-isomiRs. The size of a node linearly depends on its out-degree.

We selected colorectal cancer (TCGA-COAD) to illustrate the detailed analysis of 5′-isomiR-gene interaction network (Fig. 5). Out of 55 highly expressed isomiRs 39 molecules (71%) had at least one anti-correlated target gene. While most nodes corresponded to canonical miRNAs, four non-canonical 5′-isomiRs had high out-degrees: hsa-miR-142-3p—+1 (ITA = 34), hsa-miR-22-3p—+1 (ITA = 31), hsa-miR-203a-3p—+1 (ITA = 17) and hsa-miR-192-5p—+1 (ITA = 12). Consistent with the sequence-based target prediction, targetomes of the adjacent 5 ′-isomiRs had a little or no overlap: seven common targets of hsa-miR-203a-3p—0 and hsa-miR-203a-3p—+1 (JI = 0.17), two common targets of hsa-miR-143-3p—-1 and hsa-miR-143-3p—0 (JI = 0.29), one common target for hsa-miR-192-5p—0 and hsa-miR-192-5p—+1 (JI = 0.04), while the rest of adjacent 5′-isomiR pairs did not share target genes.

We selected two pairs of adjacent 5′-isomiR to analyze the cancer-related targeting: hsa-miR-203a-3p (—0, —+1) and hsa-miR-192-5p (—0, —+1). Interestingly, all these isomiRs showed clear tumor-suppressive patterns. Both canonical and non-canonical isomiRs targeted well-known drivers of tumor progression, including master regulators of epithelial-mesenchymal transition (EMT), such as ZEB1 and ZEB2 (see ‘Discussion’ for details).

Next, we used sets of anti-correlated target genes as an input for the functional enrichment analysis. The significant findings were regulatory interactions mediated by hsa-miR-148a-3p—0: multiple pro-cancer pathways were enriched in the set of its target genes. This included focal adhesion (KEGG hsa04510), ECM-receptor interaction (hsa04512), TGF-*β* signaling pathway (hsa04350), and PI3K-Akt signaling pathway (hsa04151). Furthermore, the mentioned pathways shared many hsa-miR-148a-3p—0 targets: COL4A1, ITGA5, ITGA11 and LAMB2 were present in three pathways, FLT1 and ROCK1 were present in two pathways, while CSF1, FBN1, LTBP1, and TGFB2 were pathway-specific. Thus, we showed that the canonical miRNA hsa-miR-148a-3p potentially suppresses colorectal tumors.

## Discussion

We developed isomiRTar—the user-friendly web portal containing information about the pan-cancer 5′-isomiRs activity. With the use of isomiRTar, researchers can promptly retrieve sequence-based target/regulator predictions and analyze co-expression profiles of putative isomiR-gene interactions in 31 types of cancers. Furthermore, the implemented pan-cancer integration of targeting information allows one to reveal “cancer-conserved” regulatory interactions supported by negative correlations of the corresponding expression values in multiple cancers.

To the best of our knowledge, two alternative isomiR web portals exist. Tumor IsomiR Encyclopedia (TIE) contains the results of pan-cancer TCGA analysis of 5′- and 3′-isomiR expression data in a web portal with multiple query and visualization modes (Bofill-De Ros et al., 2021). However, TIE lacks the possibility of isomiR target prediction and analysis. Another database, IsomiR Bank (Zhang et al., 2016), summarizes 308919 isomiRs detected from 2,727 samples with miRNA-seq, including expression data and target predictions with the miRanda tool (Betel et al., 2008). Nevertheless, the predictions available in IsomiR Bank are sequence-based and do not account for tissue or cancer specificity. Thus, we believe the developed isomiRTar portal will bring many new possibilities for the isomiR community.

The data generated by TCGA isomiR expression analysis and miRDB/TargetScan target predictions allowed us to infer several features of 5′-isomiR expression and targeting. Out of 1,022 unique 5′-isomiRs detected in the union of analyzed samples, 168 were marked as highly expressed in at least one cancer. More than half (62%) of these isomiRs had cancer-specific expression patterns, while the rest were abundantly expressed in ten or more cancer types. The high percentage of cancer-specific 5′-isomiRs agrees well with the recent study by Telonis et al. They showed that different TCGA cancers could be accurately discriminated based on the information about the “high” or “low” expression of each isomiR in a sample (Telonis et al., 2017).

Our work’s median number of sequence-based 5′-isomiR targets was 209, 95% CI [6–1082] (Fig. 2B). The previous studies reported that a typical miRNA has from 200 to 606 targets on average, depending on the target prediction strategy (Lewis, Burge & Bartel, 2005; Krek et al., 2005; Chen & Wang, 2020), which is comparable with our estimates for 5′-isomiRs. After applying cancer-specific correlation analysis the median number of genes regulated by a 5′-isomiR decreased to dozens, though this quantity termed ITA varied dramatically between cancer types (Fig. 3A). Together with the weak (though positive and statistically significant) correlation between isomiR expression and ITA, these observations justified the use of pan-cancer correlation analysis and showed the insufficiency of non-specific sequence-based target prediction.

The large-scale prediction of 5′-isomiR targets allowed us to assess the impact of 1 nucleotide variation at a 5 ′-end of an isomiR on its targetome. The median Jaccard index for the targetomes of the adjacent 5′-isomiRs was equal to 0.06, 95% CI [0.00–0.31] (Fig. 2C). Although we did not find a similar large-scale analysis in the literature, the authors of small-scale studies also reported poor overlaps between 5′-isomiRs with one nucleotide difference: JI = 0.16 for miR-411 (van der Kwast et al., 2020), JI = 0.19 for miR-9, JI = 0.28 for miR-302a (Tan et al., 2014). Note that differences between predicted targetome overlaps could be due to target prediction tools and their internal thresholds.

Aside from pan-cancer analyses, we applied isomiRTar to explore the 5′-isomiR-mediated regulatory network in colorectal cancer (TCGA-COAD project). Among eight highly expressed 5′-isomiRs, we paid special attention to miR-203a and miR-192. Both canonical and non-canonical forms of hsa-miR-203a-3p downregulated genes related to EMT: hsa-miR-203a-3p—+1 targeted transcription factor ZEB1, the master-regulator of EMT (Dongre & Weinberg, 2019), while hsa-miR-203a-3p—0 targeted ADAM12, well-known inductor of EMT (Ruff et al., 2015). A similar targeting profile was observed for hsa-miR-192-5p: the canonical form bound EMT master-regulator ZEB2 (Dongre & Weinberg, 2019) and angiogenesis stimulator WNK1 (Sie et al., 2020), while non-canonical 5′-isomiR regulated two other colorectal cancer oncogenes: NOX4 (Lin et al., 2017) and MSN (Kim et al., 2012). Another interesting finding was related to the targeting activity of hsa-miR-148a-3p—0. Specifically, functional annotation of the anti-correlated target genes list revealed strong enrichment of pathways related to adhesion, ECM-receptor interaction, the TGF-*β* signaling pathway, and the PI3K-Akt signaling pathway. We previously showed that the hypoxic microenvironment induced miR-148a downregulation in colorectal cancer cells (Nersisyan et al., 2021a), and several reports described the possible mechanisms underlying miR-148a-induced gastrointestinal cancer suppression (Li et al., 2016). These findings suggest the clear tumor-suppressive role of miR-148a in colorectal cancer. Indeed, rigorous validation of the proposed isomiR-target interactions is warranted.

Our study has several limitations. First, the used target prediction tools consider miRNA binding sites only in 3′-UTR regions of putative mRNAs. However, strong experimental evidence of miRNAs binding within coding sequences and 5′-UTRs exists (Forman & Coller, 2010; Fang & Rajewsky, 2011; Clark et al., 2014). Therefore, incorporating alternative target prediction tools that cover mRNA regions beyond 3′-UTR, *e.g.*, RNA22 (Miranda et al., 2006), is an essential direction for future isomiRTar development. Next, we note that some of the analyzed samples are heterogeneous (*e.g.*, breast tumors), and it is not possible yet to analyze different cancer subtypes separately. Inclusion of major subtypes for some cancers is also a priority feature for new versions of isomiRTar. Finally, we covered only 5′-isomiRs in our analysis since 3′-end variability does not affect the seed region of miRNAs. Yu and co-authors showed that miR-222 isomiRs differing by four nucleotides at the 3′-end play a distinct role in breast cancer (Yu et al., 2017). Though we acknowledged the existence of such studies, we decided to give preference to the conciseness of isomiRTar and did not consider 3′-end variations.

## Conclusions

In the present work, we introduce a new interactive database, isomiRTar (https://isomirtar.hse.ru), allowing researchers to analyze the interplay between 5′-isomiRs and their target genes in human cancer. The database contains the results of comprehensive bioinformatics analysis, including sequence-based 5′-isomiR target prediction and pan-cancer TCGA analysis.

##  Supplemental Information

10.7717/peerj.14205/supp-1 Supplemental Information 1 Distribution of the total number of 5′-isomiR read counts in TCGA-COAD

10.7717/peerj.14205/supp-2 Supplemental Information 2 Pan-cancer distribution of ITA values for 18 ubiquitously expressed 5′-isomiRs (highly expressed in all cancer types)

10.7717/peerj.14205/supp-3 Supplemental Information 3 The number of samples with available mRNA/miRNA-seq data and the number of highly expressed 5′-isomiRs in each used TCGA projects

## Additional Information and Declarations

### Data Availability

The following information was supplied regarding data availability: The isomiRTar portal is available at https://isomirtar.hse.ru. All source codes are available at GitHub: https://github.com/s-a-nersisyan/isomiRTar.
