# Supplementary figures and images for: isomiRTar: a comprehensive portal of pan-cancer 5′-isomiR targeting
Source: PeerJ. 2022 Oct 17;10:e14205. doi: 10.7717/peerj.14205 (PMC9583861; doi:10.7717/peerj.14205)

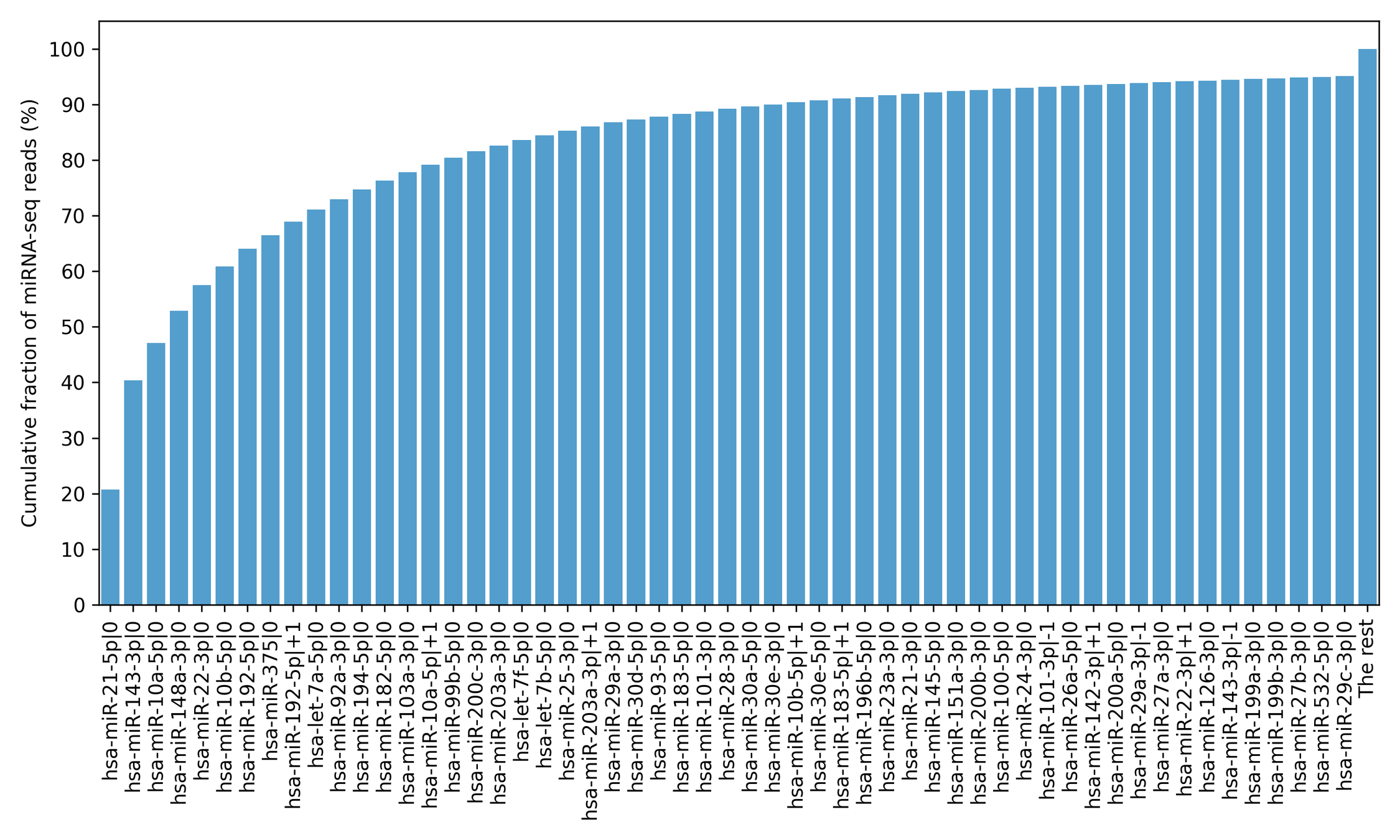

Supplement: Supplemental Information 1 [file peerj-10-14205-s001.png]

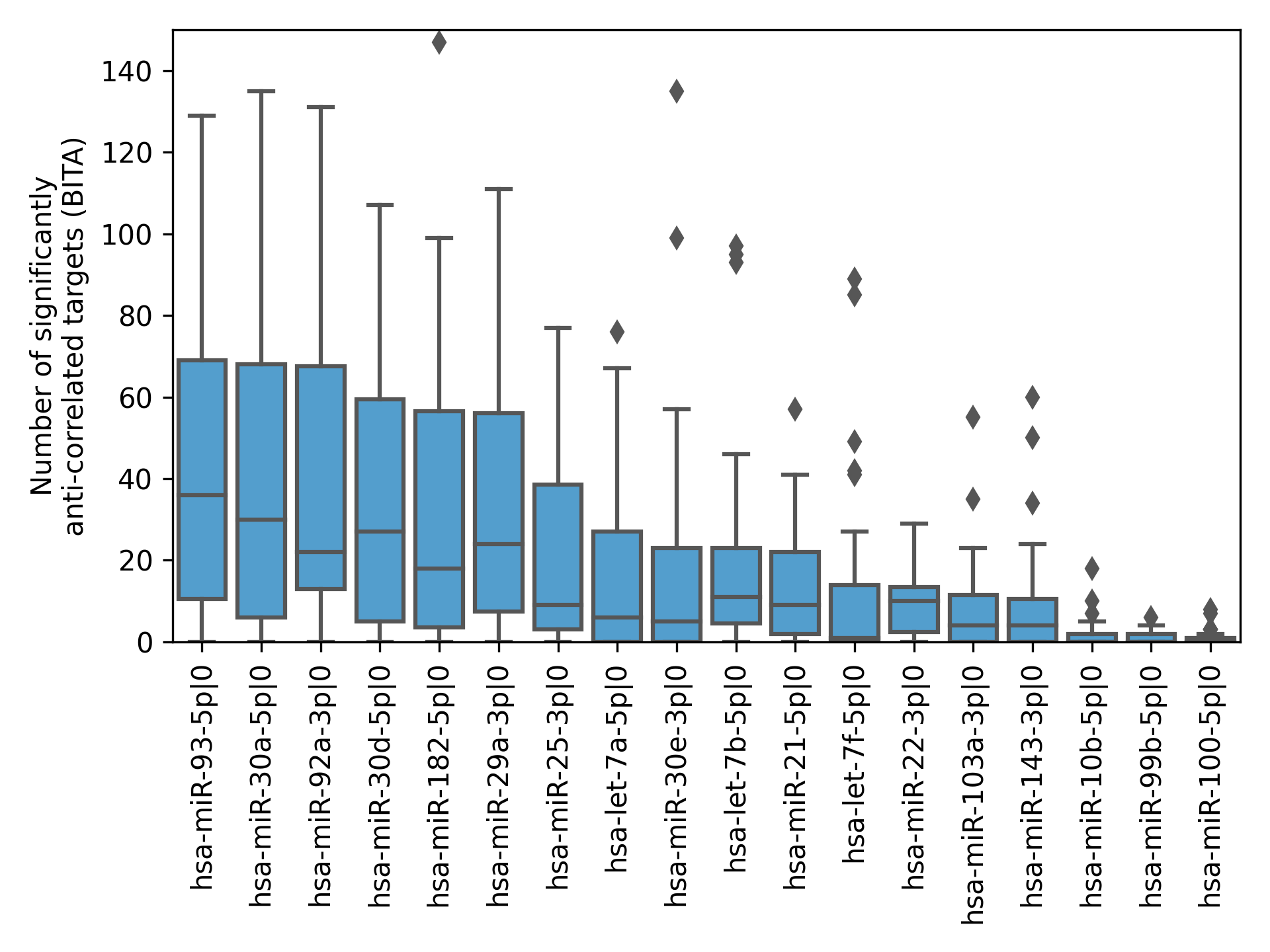

Supplement: Supplemental Information 2 [file peerj-10-14205-s002.png]
